# Does the choice of the reference model affect the results of 3D-3D superimposition procedure? A comparison of different protocols for personal identification

**DOI:** 10.1007/s00414-021-02550-x

**Published:** 2021-03-23

**Authors:** Andrea Palamenghi, Danilo De Angelis, Michaela Cellina, Chiarella Sforza, Cristina Cattaneo, Daniele Gibelli

**Affiliations:** 1 LABANOF, Laboratorio Di Antropologia E Odontologia Forense, Sezione Di Medicina Legale, Dipartimento Di Scienze Biomediche Per La Salute, Università Degli Studi Di Milano, Via L. Mangiagalli 37, 20133 Milan, Italy; 2 LAFAS, Laboratorio Di Anatomia Funzionale Dell’Apparato Stomatognatico, Dipartimento Di Scienze Biomediche Per La Salute, Università Degli Studi Di Milano, Via L. Mangiagalli 31, 20133 Milan, Italy; 3 Reparto Di Radiologia, Ospedale Fatebenefratelli, ASST Fatebenefratelli Sacco, Milan, Italy

**Keywords:** 3D-3D superimposition, Frontal sinuses, CT scan, Anatomical uniqueness

## Abstract

In literature, 3D-3D superimposition has been widely recognized as a valid method for personal identification. However, very little information is available about possible variability due to differences in protocols of registration of 3D models and calculation of RMS (root mean square) point-to-point distance. Frontal sinuses from 50 CT scans were segmented twice through the ITK-SNAP software and grouped in two samples (1 and 2). Maximum breadth, height and volume were measured. 3D models belonging to the same subject were then superimposed one on each other in 50 matches. In addition, superimposition of 50 random mismatches was performed. For each superimposition, the procedure was repeated four times choosing different reference models both for registration and calculation of RMS. Differences in RMS value among protocols of registration and RMS calculation were assessed through paired Student’s *t*-test (*p* < 0.05). Possible correlations between differences in RMS among groups and differences in frontal sinus size between the superimposed models were analysed through calculation of Pearson’s correlation coefficient (*p* < 0.05). Results showed that RMS calculation did not yield significant differences according to which 3D model is used as reference; on the other hand, RMS values from registration procedure significantly differ according to which model is chosen as reference, but only in the mismatch group (*p* < 0.001). Differences in RMS value according to RMS calculation are dependent upon all the three measurements, whereas differences according to registration protocols were significantly related only with the breadth of frontal sinuses but only in mismatches (*p* < 0.001). In no case, superimpositions of RMS values were found between matches and mismatches. This article for the first time proves that the protocol of registration and calculation of RMS significantly influences the results of 3D-3D superimposition only in case of mismatches.

## Introduction

In forensic settings, comparison of antemortem (AM) and postmortem (PM) data is essential to reach a positive identification, and usually it is performed through radiological methods. First, methods of identification were most commonly based on conventional radiography; with time, as 3D computed tomography (CT scan) was appointed as the gold standard technique for assessing head trauma and diseases by the American College of Radiology, it has been widely employed also for identification [1]. Therefore, forensic medicine benefits from the increased availability of CT scan materials which are commonly used for AM-PM data comparison and for the analysis of distinctive anatomical features that can be used for personal identification [2]. One of the most important advantages brought about by the introduction of CT scan concerns the chance of extracting a 3D model of the chosen anatomical structure, hence allowing a comparison based on the 3D surface of the anatomical structures, rather than on the mere bi-dimensional silhouette which can be appreciated through CT slice comparison, multiplanar reformation (MPR) process [3] images or conventional radiology. This type of comparison is usually performed through 3D-3D superimposition methods, where 3D models of the anatomical structures extracted from AM and PM CT scans through segmentation [4] are superimposed on each other, with quantification of the difference between the two structures [2, 5–7]. This technique represents an important improvement in comparison with traditional 2D-2D comparison usually performed on images from conventional radiology.

3D-3D superimposition techniques usually include two different procedures, i.e. the registration of the two 3D models to compare and the calculation of point-to-point distance between them. Registration consists in the movement of one of the two models to superimpose on the other one: This procedure is automatically performed by the 3D software according to the least point-to-point distance between isolated landmarks, a limited surface or even the entire surface of the 3D models [8–11]. Among these choices, the registration according to the entire surface is preferable, as it is an automatic procedure excluding the manual interaction due to the selection of reference landmarks and surfaces [2, 5].

On the other hand, the calculation of distance between two 3D models is performed through the automatic creation of a bi-univocal relationship between specific points belonging to the two models, which then results in values of maximum, minimum, mean and RMS (root mean square) point-to-point distance. These parameters are then used to discern matches and mismatches [2, 5–7].

Both the registration and point-to-point distance calculation require to choose a reference model on which the other one is superimposed and from which the distances are calculated; in their study on the validity of superimposition in orthodontic treatment, Ganzer et al. refer to the fixed model as the “master object” on which the “slave object” is superimposed [12]. For example, Beaini et al. chose to use the antemortem model of the frontal sinus as “master” and to move the postmortem model on this [13]. Although the 3D-3D superimposition techniques have been widely reported in literature, to the best of our knowledge, surprisingly no mention has been made about the possible variability of results due to the different choice of the reference model among the two compared structures, and how this variability may influence the results obtained from the identification procedure.

Given the dearth of information on which model should be used as “master” and which as “slave”, this article aims at assessing the performance of four different protocols of 3D superimposition. Namely, the study investigates the different results obtained when the registration and the calculation of the point-to-point distance are performed using both 3D models as reference. The results described here may provide help for the standardization of this novel technique of comparison and a guidance for choosing the reference model when 3D-3D superimposition is performed for personal identification.

## Materials and methods

Fifty head CT scans belonging to males aged between 20 and 82 years (mean age: 54.2 ± 17.1 years) were selected from a hospital database. Subjects affected by cranio-facial deformities, previous traumatic events, possible frontal sinusitis and cases of monolateral and bilateral agenesia of frontal sinuses were excluded. The study follows the international laws and guidelines (Helsinki Declaration) and was approved by the local ethical committee (7331/2019).

Frontal sinuses were segmented through the ITK-SNAP open-source software which allows to perform semi-automatic extraction of 3D models according to grey levels [4]. In detail, seeds are inserted within the air space of frontal sinuses, which then increase permeating the entire frontal sinus volume. The area of interest included the frontal sinuses at their respective ostium into the nasal cavities. Repeatability of the procedure of segmentation involving air spaces through a semi-automatic approach has already been verified by a previous publication [14]. Frontal sinuses from each individual were segmented twice from the same CT scan, in order to simulate AM and PM data for a possible matching procedure, and the respective models were included into two samples (named 1 and 2).

The 3D models of the frontal sinuses were then elaborated through a 3D elaboration software (VAM© software, version 2.8.3, Canfield Scientific Inc.). Maximum breadth and height of frontal sinuses were measured: Maximum height was defined as the maximum distance between the upper and the lower border of the sinus. The maximum breadth was measured between the outermost borders of the right and left sinus. In addition, volume of frontal sinuses was automatically calculated.

The 3D models underwent 3D-3D superimposition procedures two at once according to the following protocol: First, during the registration procedure, one of the two chosen 3D models was moved onto the reference one, according to the least point-to-point difference between the two surfaces; then, the RMS (root mean square) point-to-point distance of one model from the reference one was calculated. Moreover, the calculation of distance between the 3D model and the reference one provided a chromatic map of areas affected by differences in surface, coloured in blue, green and red: Blue and red colours showed the most different areas between the two models (the most and the least pronounced in comparison with the reference one, respectively), whereas the unchanged areas are reported in green.

**Fig. 1:**
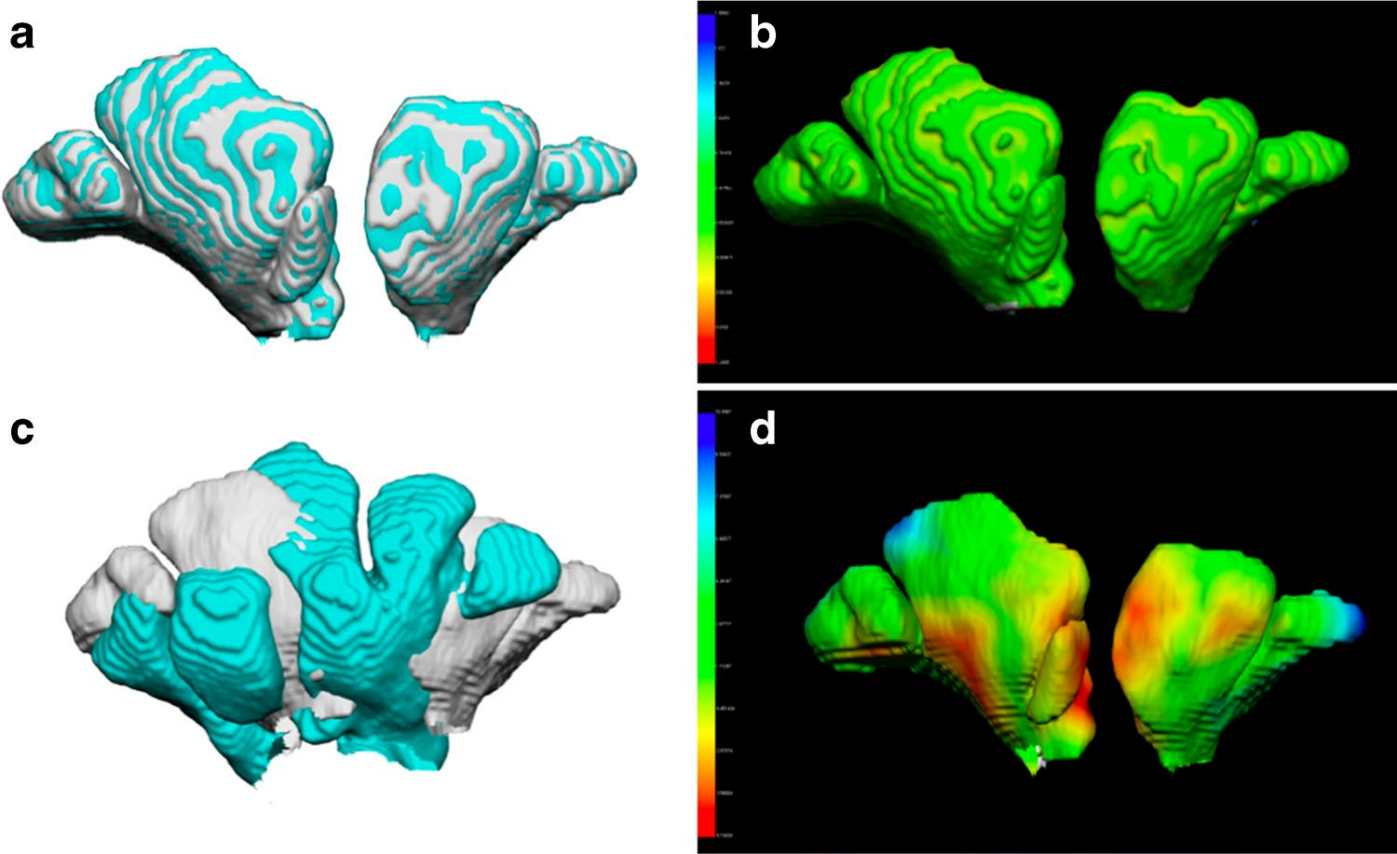
Examples of 3D-3D superimposition in a match and a mismatch. **a** Registration of two 3D models of frontal sinuses (in grey and light blue) belonging to the same individual (match). **b** Calculation of point-to-point distance between the two 3D models: The prevalence of green colour shows a wide correspondence between the two compared surfaces (RMS: 0.17 mm). **c** Registration of two 3D models of frontal sinuses (in grey and light blue) belonging to different individuals (mismatch). **d** Calculation of point-to-point distance between the two 3D models: The prevalence of colours other than green shows a discordance between the two compared surfaces (RMS: 3.10 mm)

The 3D models of frontal sinuses belonging to the same individual (in samples 1 and 2, respectively) were then superimposed: The obtained 50 superimpositions represented the group of matches (in other words, superimposition between 3D models extracted from the CT scan of the same individual). In addition, 50 superimpositions were randomly performed between 3D models of frontal sinuses belonging to different subjects from sample 1 and sample 2, representing the mismatch group (Fig. 1).

**Fig. 2:**
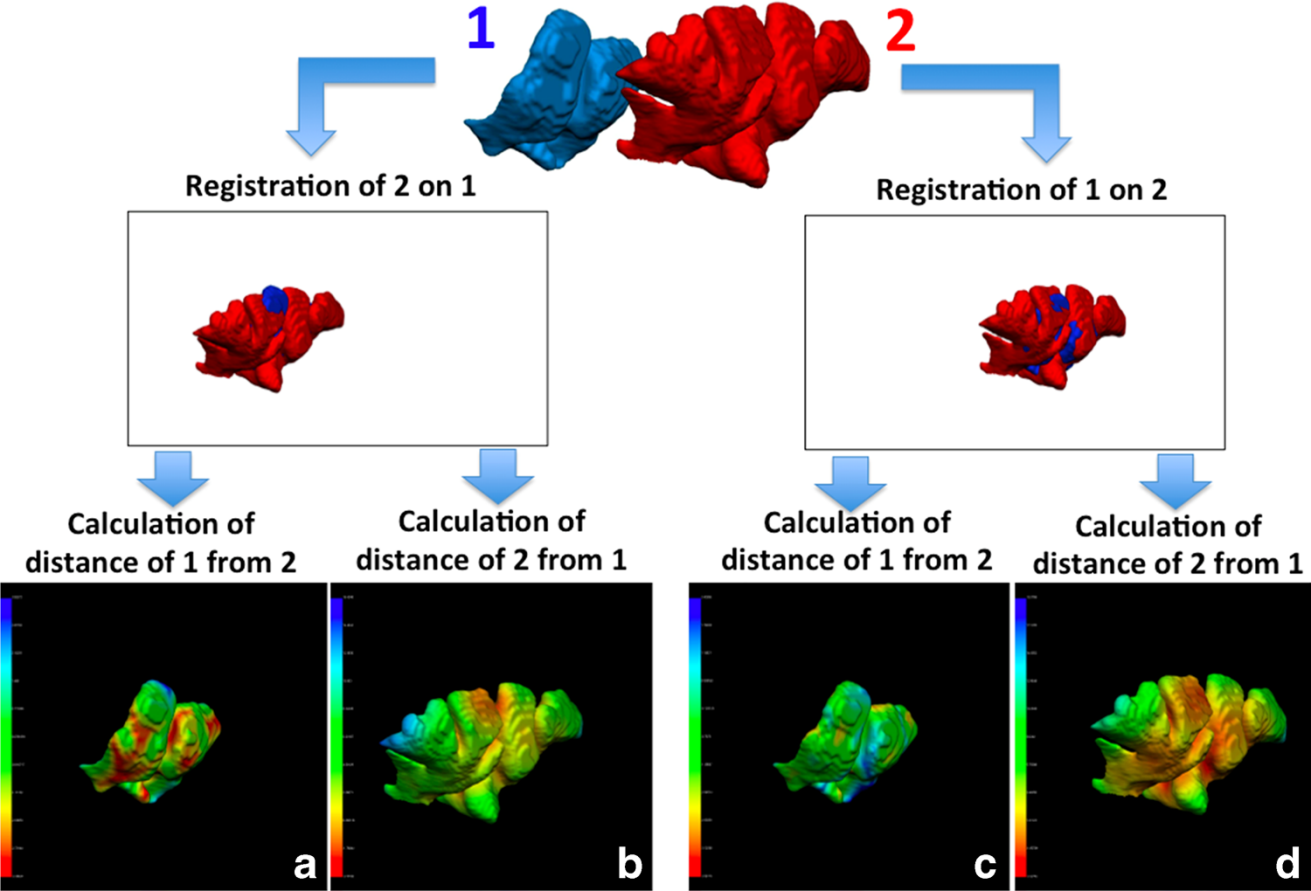
Description of the four different protocols of registration and point-to-point distance calculation compared in the present study: group **a** The 3D model from sample 1 was used as reference for registration, whereas the 3D model from sample 2 was used as reference for the calculation of point-to-point distance; group **b** The 3D model from sample 1 was used as reference both for registration and calculation of point-to-point distance; group **c** The 3D model from sample 2 was used as reference for registration, whereas the 3D model from sample 1 was used as reference for the calculation of point-to-point distance; group **d** The 3D model from sample 2 was used as reference both for registration and calculation of point-to-point distance

Every superimposition was performed four times using different models as reference for registration and calculation of point-to-point distance, both in the match and in the mismatch group, according to the following protocol (Fig. 2):

- Group A: The 3D model from sample 1 was used as reference for registration, whereas the 3D model from sample 2 was used as reference for the calculation of point-to-point distance;
- Group B: The 3D model from sample 1 was used as reference both for registration and calculation of point-to-point distance;
- Group C: The 3D model from sample 2 was used as reference for registration, whereas the 3D model from sample 1 was used as reference for the calculation of point-to-point distance;
- Group D: The 3D model from sample 2 was used as reference both for registration and calculation of point-to-point distance.

RMS values deriving from the 3D-3D superimpositions among different protocols of registration (between group A and D and between group B and C) and calculation of point-to-point distance (between group A and B and between group C and D) were analysed through paired Student’s *t*-test (*p* < 0.05). In addition, absolute value of differences in RMS between different groups (A and D, B and C for registration; A and B, C and D for calculation of point-to-point distance) was calculated, as well as the absolute value of differences in volume, breadth and height between the frontal sinuses in comparison. Possible correlations between differences in RMS among groups and differences in frontal sinus size were analysed through calculation Pearson’s correlation coefficient (*p* < 0.05).

In addition, overlapping of RMS values between matches and mismatches in the four groups was assessed to verify possible differences in identification.

## Results

On average, breadth and height of frontal sinuses were 56.9 ± 11.2 mm and 32.0 ± 7.0 mm, respectively. Volume was 8.9 ± 4.2 cm^3^.

Results of different protocols of superimpositions are shown in Table 1. On average, RMS value amounted up to 0.18 mm for matches, and between 2.26 and 2.76 mm in mismatches among different variants of registration and RMS calculation. On average, difference in RMS value was between 0.00 and 0.02 mm in matches, and between 0.41 and 1.66 mm in mismatches. On average, difference in breadth, height and volume of frontal sinuses was respectively 0.6 mm, 1.1 mm and 0.5 cm^3^ in matches (where compared 3D models from the same subject), and 12.9 mm, 6.4 mm and 2.9 cm^3^ in mismatches (where compared 3D models from different subjects).

**Table 1:** Average values and SD (standard deviation) of RMS in each group of superimposition, differences in RMS according to protocols of registration and RMS calculation and differences in breadth, height and volume of compared frontal sinuses

		RMS (mm)								|Delta| breadth (mm)	|Delta| height (mm)	|Delta| volume (cm^3^)
		Group A	Group B	Group C	Group D	Registration		RMS calculation				
						|B-C|	|A-D|	|A-B|	|C-D|			
Matches (n° 50)	Mean	0.18	0.18	0.18	0.18	0.02	0.00	0.00	0.00	0.6	1.2	0.5
	SD	0.07	0.08	0.08	0.07	0.02	0.02	0.01	0.01	0.9	1.1	0.5
Mismatches (n° 50)	Mean	2.76	2.26	2.64	2.27	0.41	0.49	1.65	1.66	12.9	6.4	2.9
	SD	1.35	1.07	1.19	1.12	0.31	0.38	1.38	1.13	7.7	4.4	1.8

Table 2 shows results of paired Student’s *t*-test among different types of protocols: RMS calculation did not yield significant differences according to which 3D model is used as reference (model from sample 1 or 2), in matches or mismatches (*p* > 0.05); on the other side, the registration procedure led to significantly different RMS values if model from sample 1 or 2 is moved onto the other one, but only in mismatch group (*p* < 0.001).

**Table 2:** *p* values obtained through paired Student’s *t*-test among different groups of superimposition in matches and mismatches. *Statistically significant values (*p* < 0.001)

		*p* values	
		Matches	Mismatches
Registration	Group B–Group C	0.308	< 0.001*
	Group A–Group D	0.117	< 0.001*
RMS calculation	Group A–Group B	0.673	0.099
	Group C–Group D	0.764	0.188

Table 3 shows the correlation between differences in RMS values according to different protocols of registration and RMS calculation, and differences in breadth, height and volume of compared frontal sinuses. Results showed that in mismatches, differences according to RMS calculation, although not significant, strongly depend upon all the three measurements (*p* < 0.001); the highest correlation coefficient was reached by breadth (0.74–0.78) followed by volume (0.62–0.72) and height (0.29–0.32). On the other side, differences in RMS values according to registration protocols were significantly related only with the breadth of frontal sinuses, again only in mismatches (*p* < 0.001), although with a low correlation coefficient (0.35–0.36).

**Table 3:** Results of Pearson’s correlation coefficient between differences in RMS among various protocols of registration and RMS calculation and differences in breadth, height and volume of compared frontal sinuses (*p* value in brackets). *Statistically significant values (*p* < 0.001)

		Matches			Mismatches		
		|Delta| breadth	|Delta height	|Delta| volume	|Delta breadth|	|Delta height|	|Delta volume|
Registration	|B-C|	− 0.124 (0.326)	0.179 (0.213)	− 0.038 (0.791)	0.359* (< 0.001)	− 0.061 (0.392)	0.107 (0.133)
	|A-D|	− 0.162 (0.262)	− 0.068 (0.638)	− 0.128 (0.377)	0.346* (< 0.001)	− 0.110 (0.122)	0.124 (0.081)
RMS calculation	|A-B|	− 0.091 (0.529)	0.006 (0.968)	− 0.107 (0.458)	0.783* (< 0.001)	0.289* (< 0.001)	0.621* (< 0.001)
	|C-D|	− 0.076 (0.601)	0.038 (0.794)	− 0.066 (0.647)	0.738* (< 0.001)	0.316* (< 0.001)	0.720* (< 0.001)

**Fig. 3:**
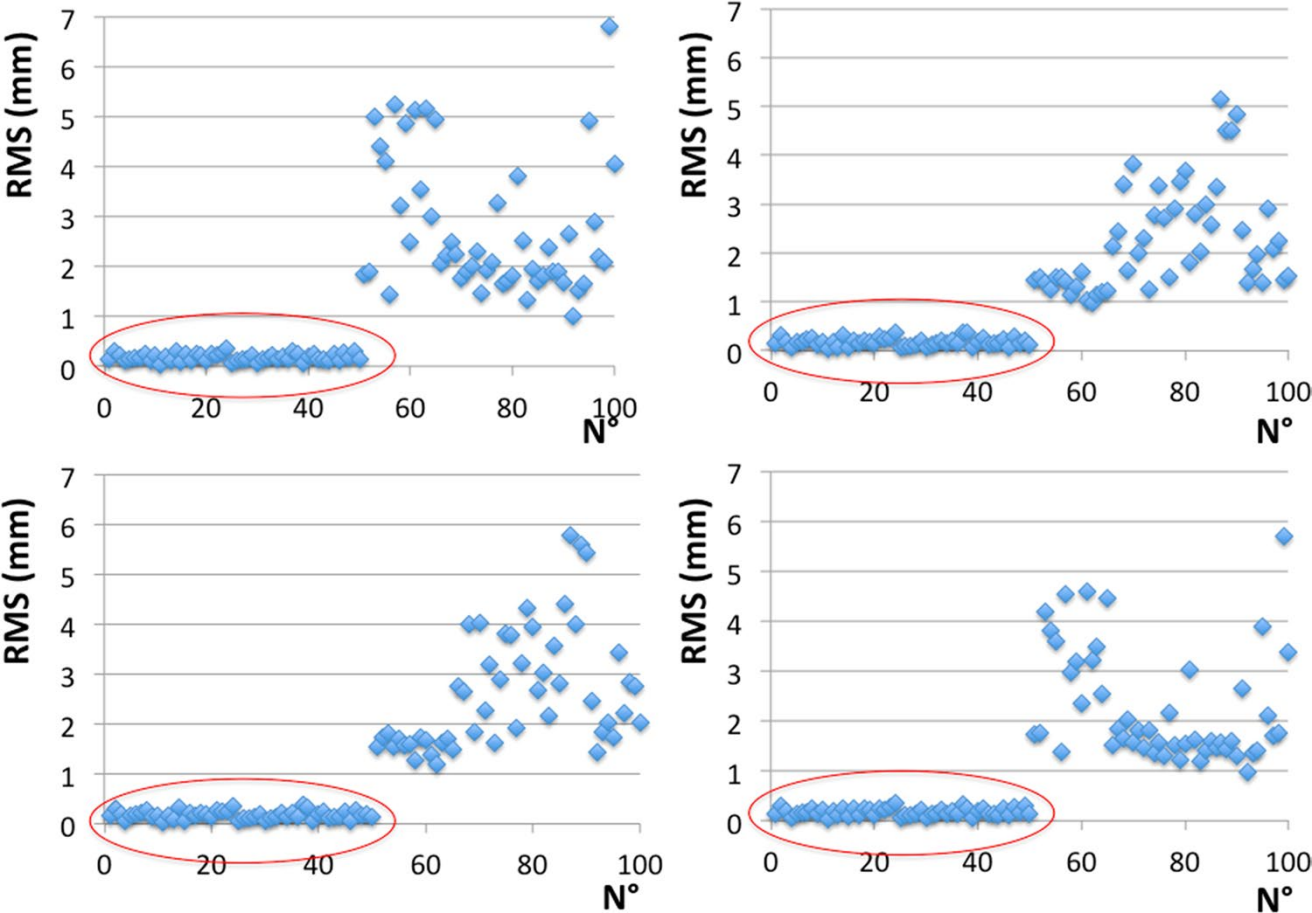
Distribution of RMS values yielded by 100 3D-3D superimpositions according to different protocols of registration and point-to-point distance calculation (from left to right, in the upper boxes A and B groups, in the lower boxes C and D); within the red ellipses are the superimpositions of matches

Finally, results from the four protocols of 3D-3D superimposition were assessed for what concerns the identification of matches and mismatches: In any group, matches and mismatches did not show any overlapping in RMS values (Fig. 3), and could be easily assessed through the threshold of 0.96 mm reported by the literature [4].

## Discussion

Superimposition of 3D models of anatomical structures has been appointed as a reliable tool in several scientific fields and applications, including evaluation of changes in growing patients [15, 16], assessment of tooth movement [8] and treatment outcome in surgery [12, 17] and management of intraoperative procedures [18]. Among the others, the forensic applications were developed as well, with special attention to the elaboration of novel methods for personal identification [2, 5–7, 13] to overcome and improve the traditional 2D-2D comparison or superimposition, usually based on images from conventional radiology [19]. According to the state of the art, 3D-3D superimposition techniques for personal identification have been applied to faces [20], teeth [7], palatal rugae [6] and frontal [5] and sphenoid sinuses [2]. Among the others, possible applications to frontal sinuses are of special interest: In fact, the size and morphological uniqueness of frontal sinuses have been extensively investigated for personal identification [2, 5, 21–25] as these paranasal sinuses are among the most diversified anatomical structures and present different configurations even in homozygotic twins [26].

Although 3D-3D superimposition has begun to be applied to the comparison of anatomical structures for personal identification, very few methodological indications are available, especially for what concerns the technical procedure: In fact, the method requires a procedure of registration followed by the calculation of point-to-point distance between two 3D models, and both these steps require to choose a reference model. Usually, literature has chosen an arbitrary reference model in 3D-3D superimpositions, but no information is available about the possible differences due to different designations [2, 5–7, 20]. However, this information is fundamental for the whole process as it presumably biases the 3D-3D superimposition procedure, potentially leading to incomparable or erroneous results.

To our knowledge, the present study is the first attempt at assessing statistical differences in RMS values in four different protocols of superimposition, according to the reference model for registration and point-to-point distance calculation. In detail, 3D-3D superimpositions were repeated four times in a group of 50 matches and 50 mismatches according to different reference models for both the steps (registration and point-to-point distance calculation): Results provided interesting data, as they showed a significant difference in RMS values according to which model is used as reference for registration. On the other hand, calculation of distances between the points of the two 3D models seems not to be significantly different if this procedure is performed choosing a 3D model rather than the other one. This result shows that registration (i.e. the preliminary movement of one model on the other one) represents the most critical step for the repeatability of 3D-3D superimposition procedures.

More interesting data were provided by the analysis of correlation between RMS differences among the four protocols and the differences in size of frontal sinuses: Although point-to-point distance calculation was not significantly different, the results strongly depend upon the difference in all measurements between the compared frontal sinuses (breadth, height and volume). This is an expected conclusion, as the 3D-3D superimposition procedure analyzes the entire surface of the model, which is strongly dependant upon the size of frontal sinuses. On the other hand, differences in RMS values between superimpositions performed using alternatively the two 3D models are dependent only from the breadth of frontal sinuses. In other words, the higher the difference in breadth between the two compared frontal sinuses, the higher the difference in RMS value obtained by choosing the two 3D models as reference.

However, although registration proved to significantly change the results of 3D-3D superimposition, each protocol was fully able to distinguish matches from mismatches, being the limits between the two groups superimposable to the threshold of 0.96 mm reported by the literature [4]. This information suggests that the 3D-3D superimposition procedure applied to frontal sinuses is a solid method for personal identification, once a standardized protocol is chosen. From this point of view, the present results suggest to use a single protocol of registration and point-to-point distance calculation and to be cautious in comparing results from different protocols, especially if they involve the registration step.

This study has clearly some limits: First, it was performed on frontal sinuses, and therefore results are strictly linked to these specific anatomical structures. Although results suggest that size of compared elements is fundamental for 3D-3D superimposition procedures, further analyses need to be performed including other structures useful for personal identification, such as palatal rugae and teeth, to confirm the present results.

Another issue concerns the specific 3D software used for the comparison: The present study used the VAM© software, provided by Canfield Scientific Inc. However, the results may vary if other 3D software are used accordingly to their specific algorithm of registration and point-to-point distance calculation. From this point of view, future studies should mandatorily focus on the comparison of 3D-3D superimposition through different software: This will give also some elements for the diffusion of 3D-3D superimposition techniques for personal identification and the possible comparison of results obtained through different methodologies.

Though apparently technical, the inferences from this study may be enormous for forensic scenarios in identification, given that the reliability of methods and their usability in court depend on the known error of the chosen method [27]; such crucial applications require strict testing of methodology, beginning precisely from registration procedures.

## Conclusion

3D-3D superimposition has proved a reliable technique for personal identification that is able to overcome the limitations of 2D images obtained by conventional radiology. However, to date, little information on the registration procedures of two 3D models has been provided and virtually no standardized protocol has been developed. This study assessed the performance of four different registration protocols and investigated the variability of results when different models are chosen as reference for registration and calculation of distance between the models. Results showed that the RMS value is affected by the choice of the reference model and the size of the anatomical structure under study, but only when frontal sinuses belonging to different individuals are compared (mismatches). In conclusion, the present article provides novel methodological information about the 3D-3D superimposition technique. Results highlight the importance of a standardized protocol and confirm the validity of this procedure for personal identification.

## Data Availability

Not applicable.

## Declarations

The study was performed according to local and international ethic rules (Helsinki guidelines), and local ethical committee (7331/2019).
